# Cost-utility of two minimally-invasive surgical techniques for operable oropharyngeal cancer: transoral robotic surgery versus transoral laser microsurgery

**DOI:** 10.1186/s12913-021-07149-x

**Published:** 2021-10-29

**Authors:** Enea Parimbelli, Federico Soldati, Lorry Duchoud, Gian Luca Armas, John de Almeida, Martina Broglie, Silvana Quaglini, Christian Simon

**Affiliations:** 1 Department of Electrical, Computer and Biomedical Engineering, University of Pavia, Pavia, Italy; 2 Department of Otolaryngology - Head and Neck Surgery, Centre Universitaire Hospitalier Vaudois, University of Lausanne, Lausanne, Switzerland; 3 Department of Otolaryngology-Head and Neck Surgery, Princess Margaret Cancer Centre- University Health Network, University of Toronto, Toronto, Canada; 4 Department of Otolaryngology - Head and Neck Surgery, Universitätsspital Zürich, University Hospital Zurich, Zürich, Switzerland

**Keywords:** Cost-utility, Transoral laser microsurgery, Transoral robotic surgery, Head and neck cancer, Oropharyngeal cancer

## Abstract

**Background:**

In the past few decades, a re-evaluation of treatment paradigms of head and neck cancers with a desire to spare patients the treatment-related toxicities of open surgery, has led to the development of new minimally invasive surgical techniques to improve outcomes. Besides Transoral Laser Microsurgery (TLM), a new *robotic* surgical technique namely Transoral Robotic Surgery (TORS) emerged for the first time as one of the two most prominent and widely used minimally invasive surgical approaches particularly for the treatment of oropharyngeal cancer, a sub-entity of head and neck cancers. Recent population-level data suggest equivalent tumor control, but different total costs, and need for adjuvant chemoradiation. A comparative analysis of these two techniques is therefore warranted from the cost-utility (C/U) point of view.

**Methods:**

A cost-utility analysis for comparing TORS and TLM was performed using a decision-analytical model. The analyses adopted the perspective of a Swiss hospital. Two tertiary referral centers in Lausanne and Zurich provided data for model quantificantion.

**Results:**

In the base case analysis TLM dominates TORS. This advantage remains robust, even if the costs for TORS reduce by up to 25%. TORS begins to dominate TLM, if less than 59,7% patients require adjuvant treatment, whereby in an interval between 55 and 62% cost effectiveness of TORS is sensitive to the prescription of adjuvant chemoradiation therapy (CRT). Exceeding 29% of TLM patients requiring a revision of surgical margins renders TORS more cost-effective.

**Conclusion:**

Non-robotic endoscopic surgery (TLM) is more cost-effective than robotic endoscopic surgery (TORS) for the treatment of oropharyngeal cancers. However, this advantage is sensitive to various parameters, i.e.to the number of re-operations and adjuvant treatment.

## Background

Within the last 15 years efforts were made to reduce treatment-related toxicity of head and neck cancer surgery through the development of new minimally invasive surgical techniques. Besides Transoral Laser Microsurgery (TLM), a new *robotic* surgical technique namely Transoral Robotic Surgery (TORS) emerged for the first time as one of the two most prominent and widely used minimally invasive surgical approaches. This surgical method was found particularly useful for the treatment of oropharyngeal cancer, a sub-entity of head and neck cancers [1–3].

TORS is a technique that utilizes wristed robotic surgical technology through a transoral approach in order to facilitate en-bloc resection of tumors. Resections are performed using electrocautery with endoscopes providing different angles of visualization. This technique provides excellent visualization of the disease and possibility to resect the tumor in one piece allowing for more precision in terms of margin analysis [4].

TLM, on the other hand, utilizes lasers, and visualization through laryngoscopes and mouth gags for exposure. Typically, the field of vision is smaller than with TORS, thus the laryngoscopes have to be repositioned several times during the intervention. TLM follows the philosophy of resecting the tumor in pieces. This may confer a better control over the deep margin as a consequence of traversing the tumor and assessing the different cauterisation characteristics between tumor and normal tissue, it may add however a certain degree of uncertainty to the final reading of the margins [5].

A recent population-level analysis demonstrated equivalent survival and similar positive margin rates with both techniques, but a significantly higher rate of post-operative chemoradiation in the TLM group suggesting that uncertainties over margins may lead treating physicians to rather favour the more aggressive postoperative treatment [6].

TLM appears to have a steeper learning curve than TORS. This is of importance to head and neck cancer programs wanting to implement one or the other technique. To the contrary, TLM infers fewer total costs as a consequence of the high equipment and disposable costs incurred during robotic surgery [7].

In summary, a comparative analysis of the two minimally-invasive techniques is warranted for which we used a decision-analytic model for comparing TORS and TLM from the cost-utility (C/U) point of view. Despite TLM and TORS having been independently compared to non-surgical treatment for head and neck cancers [8–14], to the best of our knowledge, there is no literature directly comparing TORS and TLM.

## Materials and methods

Our base case consists of a Swiss patient with an oropharyngeal squamous cell carcinoma (OPSCC), age 55, with operable T-category (T1 or T2) OPSCC and a probability of positive nodal disease (N+) between 60 and 70%. Our analyses are performed from a Swiss hospital perspective and with a lifetime horizon (life expectancy for the male Swiss population is 81.9 years of age according to data from the Swiss O*ffice Federal de la Statistique -* FSO).

We developed a two-stage model based on (i) a published model about the economic evaluation of TORS vs radiotherapy [12], (ii) additional literature [6] and (iii) authors’ expertise and statistics from two Swiss tertiary referral centers.

The first-stage decision tree accounts for short-term outcomes of the surgery and its complications which are, in turn, carried forward as initial conditions for a second-stage model representing long-term outcomes through a Markov process.

**Fig. 1:**
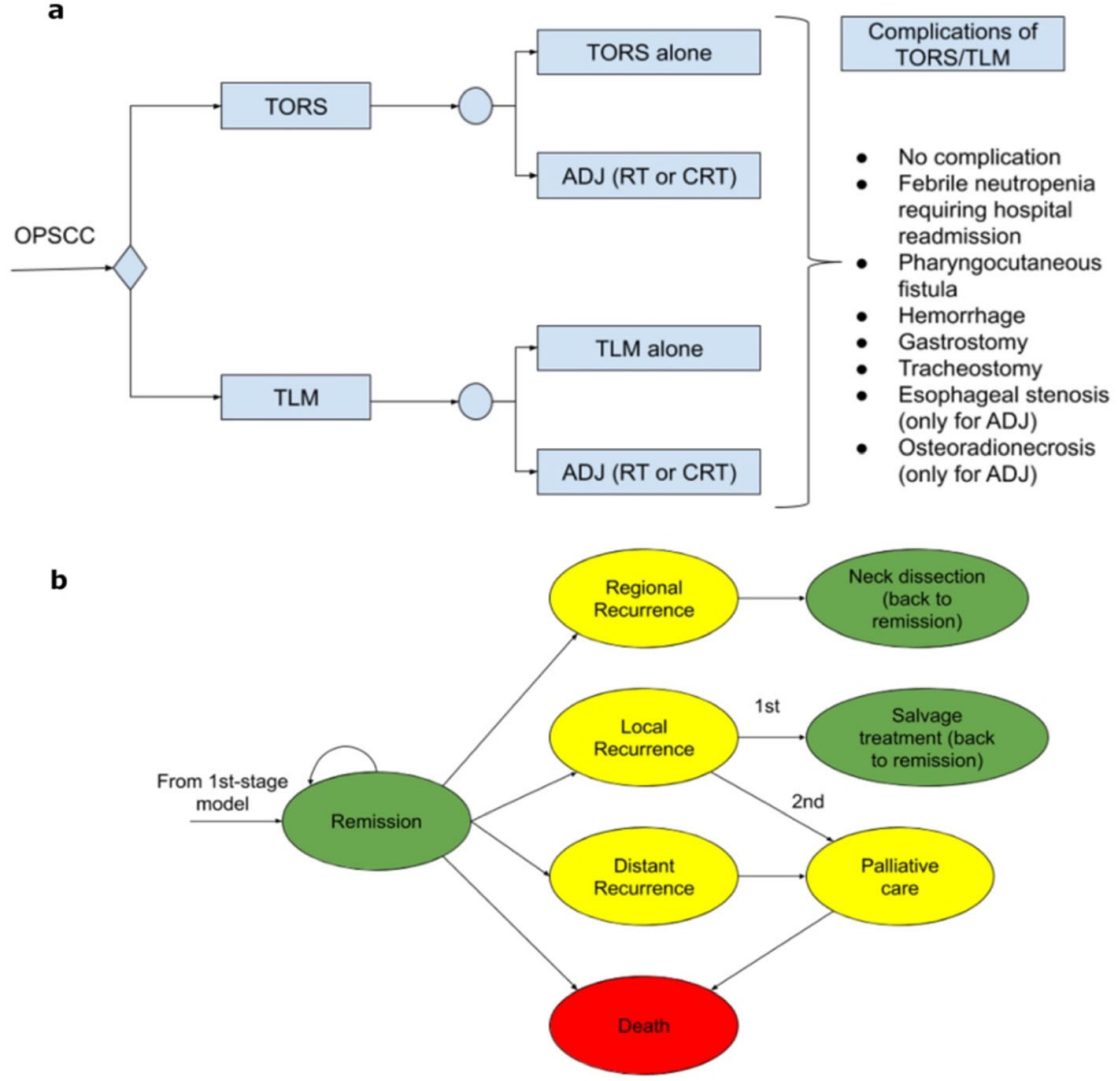
Short-term outcomes decision tree (**a**) and second-stage Markov model (**b**)

The first-stage model is depicted in Fig. 1. The two surgical strategies constitute alternatives for the first decision node, after which a chance node distinguishes between cases undergoing surgery alone, and cases requiring adjuvant radiotherapy (RT) or chemoradiotherapy (CRT). Finally, potential complications of the surgical interventions and, where appropriate, associated adjuvant therapy are modeled.

The second-stage model deals with long-term outcomes and is constituted by a Markov model (Fig. 1b). It represents patients entering a state of remission after treatment and models their transitions through other possible health states until death. The Markov cycle has been set to 3 months and the time horizon is the entire patient life. Initial rewards of each Markov model are carried forward from the results of the first-stage model.

Model parameters representing estimates of probabilities of adjuvant treatment were derived from data published by Li et al., which constitute the largest and most recent published database study with relevant outcome data. Other parameters modeling clinical events such as complication rates and recurrence rates were determined from systematic review of the literature [15]. The hospital admission rate for CRT was set at 75% of patients to be admitted once, and 25% twice. The proportion of patients needing hospital admission for RT was set at 25% only once. Regarding the need for a gastrostomy we considered a percutaneous endoscopic gastrostomy (PEG)-rate for chemoradiation (CRT) of 70% while 20% for radiation therapy (RT) as per institutional data from the Centre Hospitalier Universitaire Vaudois (CHUV) and the Universitätsspital Zürich (USZ).

Transition probabilities between different health states of the Markov models were directly adopted from de Almeida et al. [12] and no relevant difference in survival is assumed between the TORS and TLM arms of the model, based on a recent retrospective analysis of the National Cancer Data Base (NCDB) [16]. Table 1, probabilities of events section, reports the specific value used for each parameter. Risk of death from non-cancer-specific causes is modeled following Swiss life tables, acquired through the FSO.

**Table 1:** Model parameters: Probabilities of occurrence of events, costs and utilities

Variable name	Description	Mean	Standard deviation	Distribution type	Parameter 1 (alpha)	Parameter 2 (beta)
**Probabilities of events**						
*pes*	Probability of esophageal stenosis	0.0476	0.0005	Beta	4	80
*phem*	Probability of hemorrhage	0.0243	0.0001	Beta	6	241
*pho_adj*	Probability of hospital readmission after adjuvant	0.1731	0.0027	Beta	9	43
*pho_s*	Probability of hospital readmission (TORS or TLM)	0.0333	0.0010	Beta	1	29
*plg*	Probability of long-term gastrostomy (1 year) after adjuvant treatment	0.0500	0.0003	Beta	9	171
*plt*	Probability of long-term tracheostomy (1 year)	0.0226	0.0001	Beta	4	173
*psg*	Probability of short-term (6 months) gastrostomy (TORS or TLM)	0.0144	0.0001	Beta	2	137
*psg_adj*	Probability of short-term (6 months) gastrostomy after adjuvant	0.2991	0.0019	Beta	32	75
*por*	Probability of osteoradionecrosis	0.0265	0.0002	Beta	4	147
*ppf*	Probability of pharyngocutaneous fistula	0.0253	0.0001	Beta	10	385
*pTLMAlone*	Probability of TLM alone	0.4085	0.0007	Beta	134	194
*pTorsAlone*	Probability of TORS alone	0.3740	0.0001	Beta	824	1379
*pCRT_TLM*	Probability of adjuvant CRT (TLM)	0.6289	0.0012	Beta	122	72
*pCRT_tors*	Probability of adjuvant CRT (TORS)	0.5272	0.0002	Beta	727	652
*pRT_TLM*	Probability of adjuvant RT (TLM)	0.3711	0.0012	Beta	72	122
*pRT_tors*	Probability of adjuvant RT (TORS)	0.4728	0.0002	Beta	652	727
*plr*^*a*^	Probability of local or regional recurrence (first 2 years)	0.0064	0.0000	Beta	11	1715
*prr*^*a*^	Probability of regional recurrence (first 2 years)	0.0064	0.0000	Beta	11	1715
*pdr*^*a*^	*Probability of distant recurrence (first 2 years)*	*0.0038*	*0.0000*	*Beta*	*11*	*2900*
**Costs (CHF)**						
*cTORS*	*Cost of TORS*	*14,739*	*869.31*	*Gamma*	*287.4635*	*0.0195*
*cTLM*	*Cost of TLM*	*12,671*	*516.23*	*Gamma*	*602.4698*	*0.0475*
*cCRT*	Cost of adjuvant CRT	33,911	2079.08	Gamma	266.0350	0.0078
*cRT*	Cost of adjuvant RT	27,962	1714.35	Gamma	266.0342	0.0095
*cES*	Cost of esophageal stenosis	2362	410.65	Gamma	33.0832	0.0140
*cGAST*	Cost of gastrostomy	4332	410.65	Gamma	111.2820	0.0257
*cHR_adj*	Cost of hospital readmission (for adjuvant)	10,097	619.05	Gamma	266.0342	0.0263
*cHR_s*	Cost of hospital readmission (TORS or TLM)	8203	803.41	Gamma	104.2498	0.0127
*cORN*	Cost of osteoradionecrosis	32,111	1077.71	Gamma	887.7769	0.0276
*cPF*	Cost of pharyngocutaneous fistula	82,892	333.96	Gamma	61,609.3654	0.7432
*cPH*	Cost of hemorrhage (from surgical site)	4469	415.50	Gamma	115.6865	0.0259
*cTRACH*	Cost of tracheostomy	11,688	612.67	Gamma	363.9366	0.0311
*cREM*	Cost of remission 0–2 y	168.5	9.68	Gamma	303.2588	1.7998
*c2REM*	Cost of remission 2–5 y	60	9.68	Gamma	38.4518	0.6409
*cPC*	Cost of palliative care	4137	367.86	Gamma	126.4754	0.0306
*cRR*	Cost of regional recurrence	7047	464.24	Gamma	230.4227	0.0327
*cLR_chemorad*	Cost of local recurrence (chemoradiation)	34,041	2079.08	Gamma	268.0786	0.0079
*cLR_s*	Cost of local recurrence (surgical resection)	40,513	2050.27	Gamma	390.4511	0.0096
*cDM*	Cost of distant metastasis	4137	367.86	Gamma	126.4754	0.0306
*cPanendo*	Cost of panendoscopy	388	23.79	Gamma	266.0337	0.6857
**Utilities**						
*uSURG*	Utility coefficient of TORS or TLM	0.902	0.203	Beta	1.0328	0.1122
*uRT*	Utility coefficient of adjuvant RT	0.850	0.275	Beta	0.5831	0.1029
*uCRT*	Utility coefficient of adjuvant CRT	0.794	0.317	Beta	0.4984	0.1293
*uHR*	Utility coefficient of hospital readmission	0.954	0.140	Beta	1.1820	0.0570
*uPF*	Utility coefficient of pharyngocutaneous fistula	0.932	0.194	Beta	0.6374	0.0465
*uPH*	Utility coefficient of postoperative hemorrhage	0.910	0.203	Beta	0.8986	0.0889
*Ug*	Utility coefficient of gastrostomy	0.916	0.209	Beta	0.6975	0.0640
*Ult*	Utility coefficient of long-term tracheostomy	0.852	0.271	Beta	0.6109	0.1061
*ues*	Utility coefficient of esophageal stenosis	0.826	0.284	Beta	0.6459	0.1361
*uORN*	Utility coefficient of osteoradionecrosis	0.791	0.302	Beta	0.6428	0.1698
*urem*	Utility coefficient of remission after surgery and adjuvant	0.980	0.099	Beta	0.7702	0.0346
*uremonlysurg*	Utility coefficient of remission after TORS or TLM alone	0.957	0.151	Beta	0.9798	0.0200
*ureg*	Utility coefficient of regional recurrence	0.859	0.283	Beta	0.4401	0.0722
*ulocxrt*	Utility coefficient of local recurrence, RT	0.771	0.302	Beta	0.7216	0.2143
*uloc*	Utility coefficient of local recurrence, requiring surgery	0.755	0.316	Beta	0.6436	0.2088
*udist*	Utility coefficient of distant recurrence	0.213	0.336	Beta	0.2262	0.5106
*upall*	Utility coefficient of palliative care	0.307	0.350	Beta	0.1033	0.3816

^a^NOTE: for prr, plr and pdr 80% of recurrences were modeled in the first 2 years, and the remaining 20% between 2 and 5 years posttreatment (probabilities were adjusted accordingly, assuming 5% of patients have recurrences in the first 2 years^6^)

Costs were directly acquired from the Centre Hospitalier Universitaire Vaudois and the Universitätsspital Zürich administrative departments. Having adopted a hospital perspective, costs incurred by the patient are not considered in our analyses. All costs are represented as Gamma distributions, as suggested by Huinink et al. [17] for variables with values greater or equal than 0 (Table 1). An equal discounting rate of 3% has been employed for both costs and effects incurred in the future .

Utility coefficients (UCs) for the health states included in the model were collected with Standard Gamble method through our UceWeb [18, 19] platform from a set of 41 Swiss healthy volunteers. Eighteen different scenarios were evaluated by each participant [20]. Rating Scale method was also administered, to familiarize participants with the tool and as a consistency check of the obtained values. As for probabilities, UCs are represented as beta distributions (Table 1).

Willingness-to-pay was set to 4000 Swiss Franc (CHF)^1^/Quality associated life months (QALM), i.e., 48,000 Swiss Franc (CHF)/Quality associated life years (QALY) [21, 22]. Incremental cost was computed from the difference in expected cost (CHF) between TORS and TLM. Similarly, incremental utility was computed from the difference in expected utility between TORS and TLM. The incremental cost-utility ratio was derived taking the quotient between incremental cost and incremental utility. All cost-utility analyses were performed using TreeAge Pro 2019 software (Williamstown, MA, 2019).

Key model parameters were varied using one-way and two-way deterministic sensitivity analysis in order to assess their impact on the results. In particular, we explored the key role of adjuvant therapy (RT or CRT) after surgery and costs of treatment. In order to perform probabilistic sensitivity analysis (PSA) all parameters were represented using probability distributions. Probabilities of event occurrence were represented as beta distributions, as indicated for variables ranging from 0 to 1 [17] (Table 1). Probabilistic sampling was performed from the distributions described above for probabilities (Beta), costs (Gamma) and utilities (Beta). PSA was performed using second-order Monte-Carlo simulations using 1000 simulations. Incremental cost and effectiveness were plotted with 95% confidence ellipsoids.

## Results

The base case analysis used model parameters presented in the methods section and Table 1. Results in Table 2 show that TORS is moderately more effective than TLM (Months: 342.72 versus 342.62) but also more costly than TLM (Costs in CHF 56879.13 versus 53,518.28). When taking into account quality of life, TLM dominates TORS with slightly higher QALMs (216.40 versus 216.31) at a lower cost.

**Table 2:** Base case analysis results

	TORS	TLM
Months	**342.72**	342.62
QALMs	216.31	**216.40**
Cost (CFH)	56,879.13	**53,518.28**

**Fig. 2:**
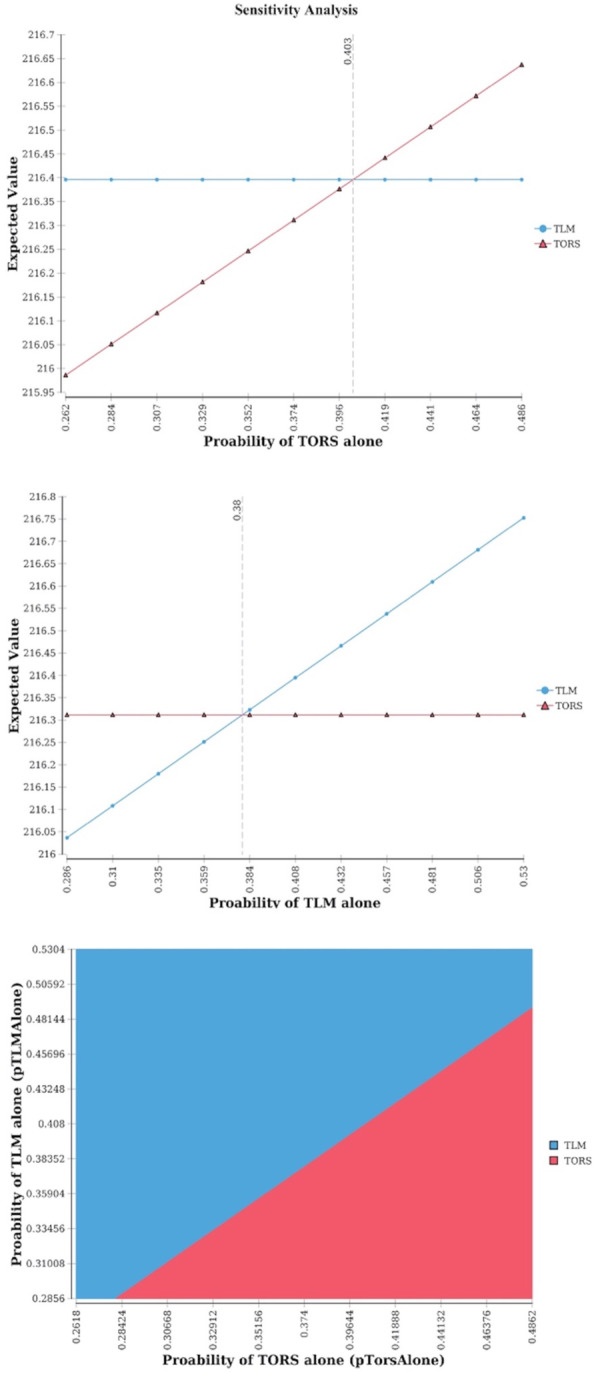
1-way and 2-way sensitivity analyses of probability of TORS alone and probability of TLM alone. NOTE: Higher expected value in one-way analyses corresponds to the winning strategy. Similarly, the color of the area containing a specific point in the 2-way analysis plane identifies the winnng strategy (i.e. dominating or with a higher utility and an ICUR < threshold)

An important role is assumed by the adoption of adjuvant (chemo) radiotherapy after TORS and TLM. Univariate sensitivity analyses on effectiveness show that when the probability of adjuvant therapy is less than 1–0.403 (< 0.597) for TORS, TORS is the optimal option. The same is true for TLM when the probability of adjuvant therapy is less than 1–0.38 (< 0.62). When varied simultaneously in a 2-way sensitivity analysis the effect on the optimal strategy is also evident, as the modality with a lower chance of needing adjuvant treatment is preferred by the model (Fig. 2).

**Fig. 3:**
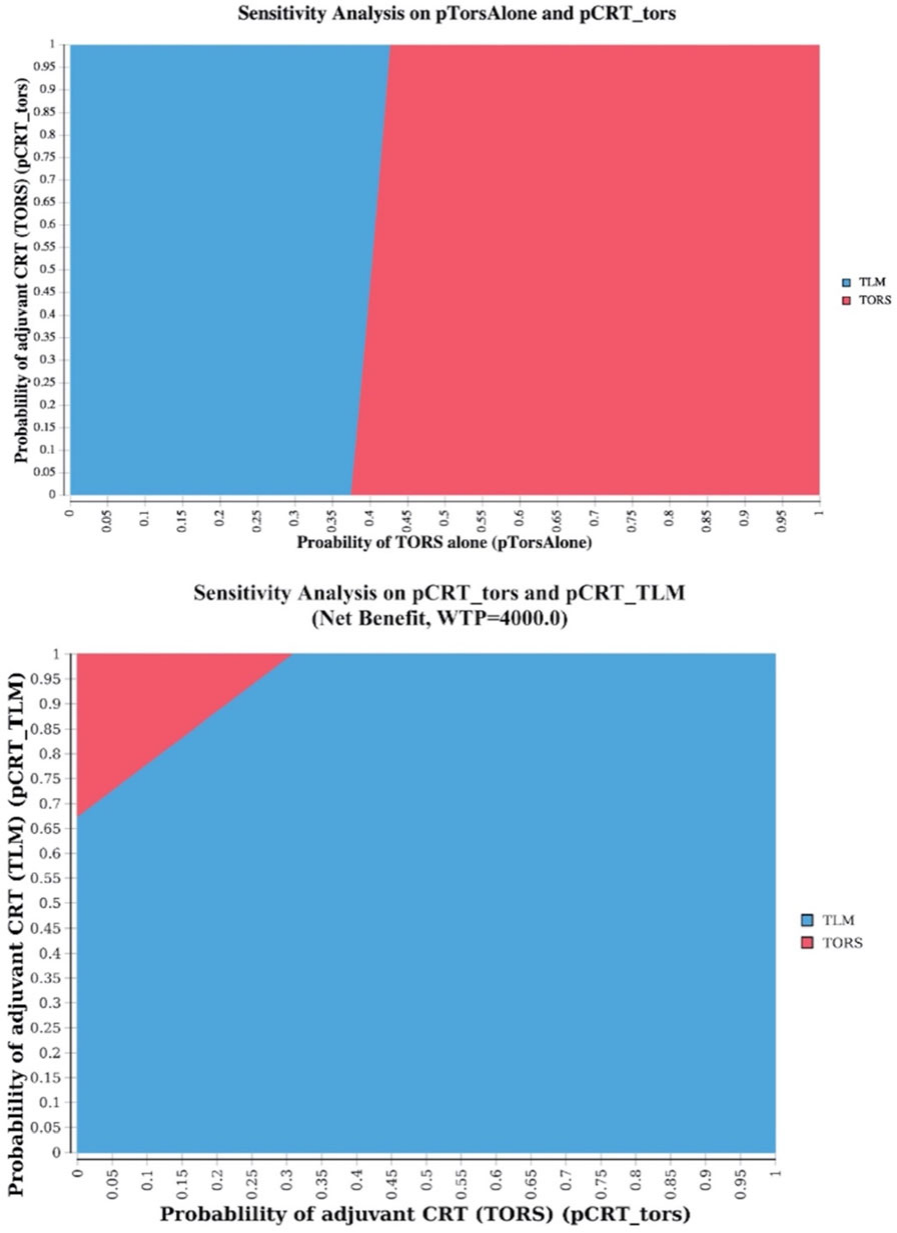
2-way sensitivity analyses for pTorsAlone and pCRT_Tors, and probability of adjuvant chemoradiotherapy after TORS and after TLM NOTE: the color of the area containing a specific point in the 2-way analysis plane identifies the winnng strategy (i.e. dominating or with a higher utility and an ICUR < threshold)

In the hypothetical scenario where improvements to TORS, or careful patient selection, vary the proportion of patients needing adjuvant therapy (i.e., pTorsAlone varies) also the type of the adjuvant treatment begins to play a role. Decreasing the proportion of patients that receive chemoradiotherapy, instead of radiotherapy, as adjuvant treatment after TORS, can also make TORS a preferred option over TLM. A 2-way sensitivity analysis on the probability of adjuvant CRT after TORS (pCRT_Tors) and the probability of TORS-only (pTorsAlone) shows that if the use of adjuvant therapy is lower than 1–0.55 (< 0.45), then TORS is the preferred option. However, if the use of adjuvant therapy after TORS is between 0.55–0.62 (pTorsAlone is in the [0.38–0.45] interval) the proportion of patients receiving CRT as adjuvant therapy has to stay under a certain value for TORS to be the optimal alternative (Fig. 3).

A 2-way sensitivity analysis (Fig. 3) shows how being able to decrease the proportion of patients that receive chemoradiotherapy, instead of radiotherapy alone, can make TORS a preferred option over TLM. It shows how the combination of probabilities of adjuvant CRT after TORS (pCRT_TORS) and of adjuvant CRT after TLM (pCRT_TLM) that sees TORS the preferred option is rather small.

**Fig. 4:**
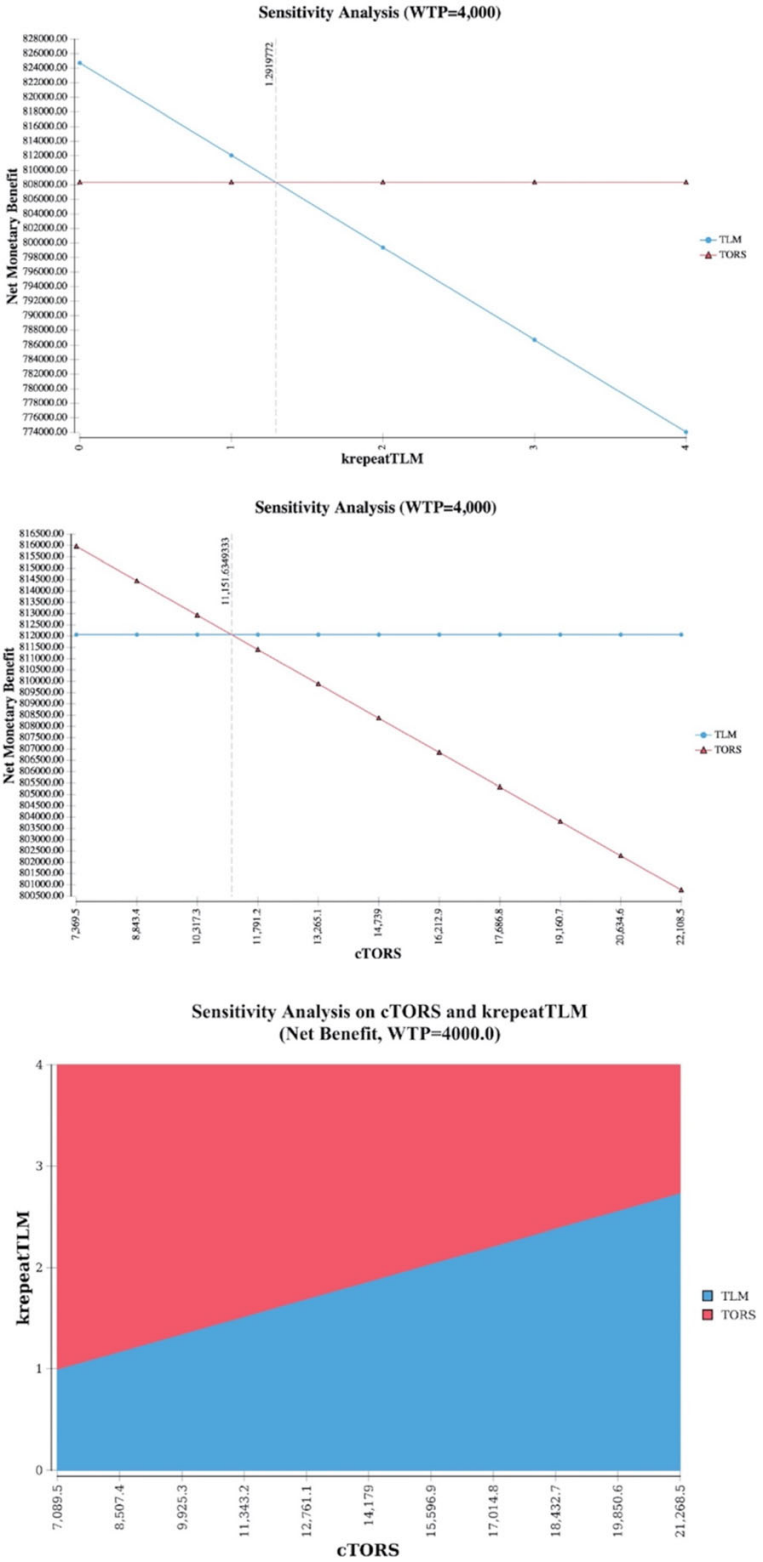
1 way sensitivity analysis on cost of TLM (based on number of re-resections needed for negative margins), cost of TORS, and 2-way sensitivity analysis combining the two. NMB = WTP*QALMs. NOTE: Higher expected value in one-way analyses corresponds to the winning strategy. Similarly, the color of the area containing a specific point in the 2-way analysis plane identifies the winning strategy (i.e. dominating or with a higher utility and an ICUR < threshold)

Given that during TLM the tumor is resected in multiple pieces [23, 24], it is common in clinical practice to bring back patients for one or multiple resections in order to achieve negative margins [25]. Our analysis shows that as soon as TLM needs to be repeated once in more than 29% of the patients (krepeatTLM > = 2, threshold value = 1.292 in sensitivity analysis), the increased cost compared to TORS makes TORS the preferred option with a higher net monetary benefit (Fig. 4). On the other hand, results of the base case analysis are rather robust to changes in cost of TORS, confirming TLM as the optimal option even for a relevant (~ 24%) decrease in TORS cost. A 2-way sensitivity analysis highlights, how repeating TLM even only once (krepeatTLM = 2) and keeping TORS cost as-is, results in TORS being the preferred option for cost-effectiveness (Fig. 4).

**Fig. 5:**
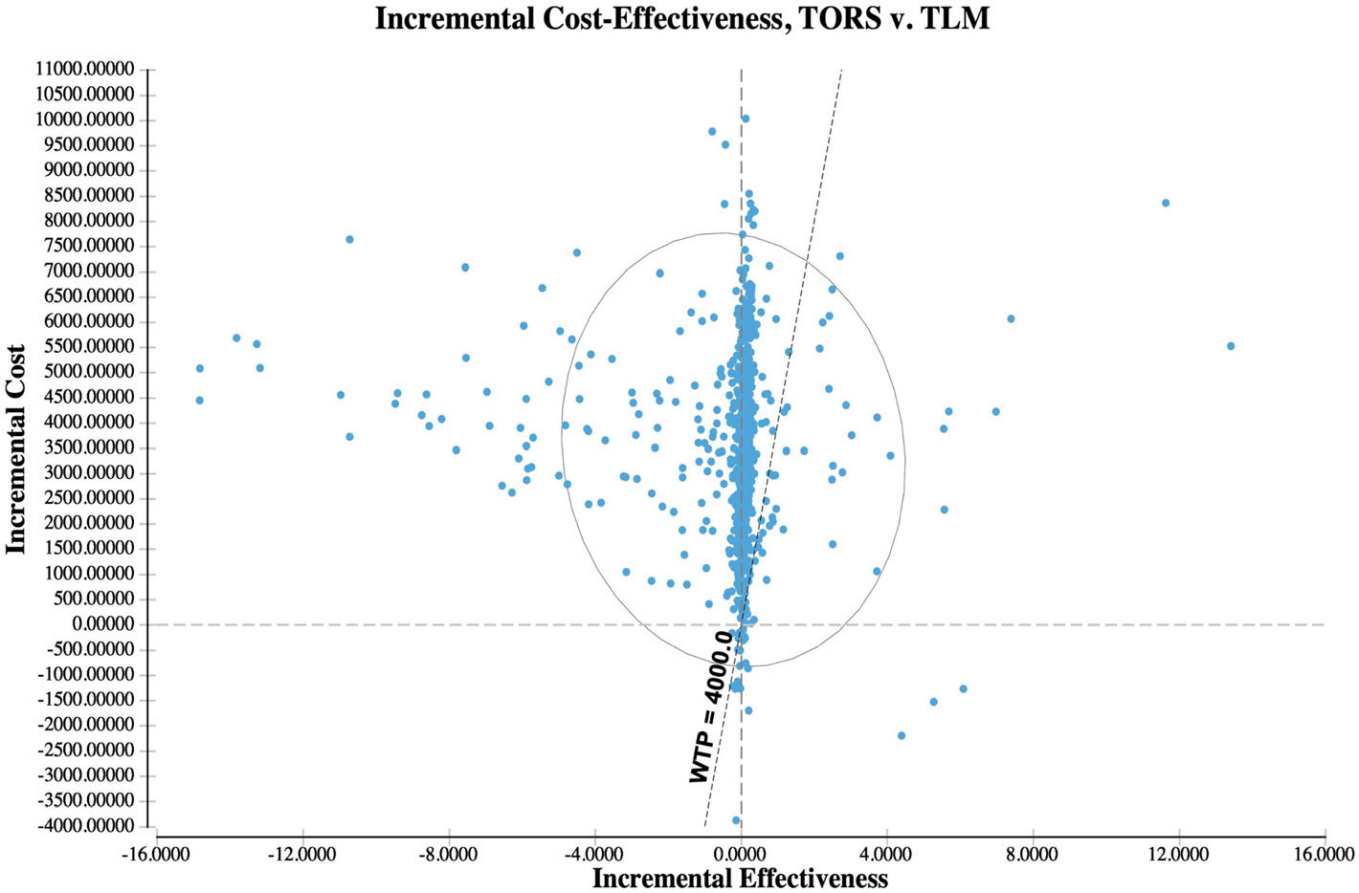
PSA, base case analysis. The simulation has been performed on 1000 samples. 95% confidence ellipsoid and willingness to pay line are also reported in the figure

Using base-case parameters, PSA shows most simulations favoring TLM, having lower cost (incremental cost is > 0 for most simulations) and close to 0 incremental effectiveness compared to TORS. In the 1000 simulations, with a willingness to pay of 4000 CHF/QALM (i.e. 48,000 CHF/QALY), TORS dominates in only 1.1% of the cases, is cost-effective in 5.2%, while TLM is cost-effective in 66% of the cases, and dominates in the remaining 27.7% (Fig. 5).

## Discussion

Our two-stage model-based analysis shows that TLM is currently a cost-effective surgical treatment choice for operable OPSCC. Results, albeit robust, are sensitive to a variability in the proportion and nature of adjuvant therapy and need of performing TLM re-resections impacting costs.

TORS becomes more cost-effective than TLM for low rates of post-operative adjuvant treatment (RT or CRT). The choice of the optial strategy is also sensitive to the type of adjuvant treatment, where high rates of RT are faivored. Thus, higher rates of postoperative therapy after TORS reduce its overall utility and suggest that a careful case selection, in particular limiting cases needing adjuvant CRT, might be important to impact its cost-effectiveness.

The potential need to repeat TLM surgery for close or positive margins, even only once, results in a significant change in TLM costs, favoring TORS as the cost-effective alternative. According to our one-way sensitivity analysis on costs of TLM (Fig. 4) the threshold for TORS to be as cost-effective as TLM based on the number of re-interventions is found to be 1.29, suggesting that if 29% or more patients require re-resections after TLM, and given that none of the TORS patients need to be taken back to the operative room outside the regular setting used for the base case scenario, TORS is superior in terms of cost-effectiveness. This percentage is already reached in certain centers even with large experience according to current literature. In a study comprising of 1467 patients treated with TLM for cancers of the oral cavity, oropharynx, larynx, and hypopharynx, 386 patients (26,3%) were taken back for a second resection, and of those even another 22 for a third and a fourth resection with TLM [25]. It seems therefore critical to avoid second operations with TLM by eventually relying more on the use of frozen sections whenever and wherever feasible.

In general, advantages of TORS are the learning curve, allowing for easier adaptation of the surgeon and better results in a shorter amount of time [26, 27]. A clear disadvantage are the upfront capital costs that are widely exceeding the costs of TLM [7]. TLM to the contrary has lower upfront costs, but is technically more challenging requiring more training and a longer time until mastering the procedure [26].

There are certainly limitations of this type of analysis. Modeling is based on various parameter estimates, most of which are retrospectively taken from various sources. Also, this analysis has been performed from a Swiss hospital perspective. It is probable that other healthcare systems account for other costs eventually limiting the generalizability of the results. Moreover, it is worth mentioning that there are two main ways of conducting cost-utility analyses. One is through economic modeling (used in this paper) and the other one is to employ statistical analysis directly on data from clinical trials where the two competing strategies (TORS vs TLM) are compared. One argument in favor of the use of modeling is to extrapolate beyond the often limited follow-up period of clinical trials. This is particularly relevant in our case since we adopted a lifetime temporal horizon in our study. Another argument is the possibility of using a synthesis of evidence coming from several studies (e.g., through systematic reviews and meta-analyses) when building and quantifying the decision-analytical model, with the potential of improving generalizability of the results. Finally, a decision-analytic model allows to perform sensitivity analysis in order to answer those “what if” questions that are useful for gaining a deeper insight in the problem. For example, in our study, we detected which are the conditions that would make TORS more cost/effective than TLM. However, approaches based on modeling are also limited by the unavoidable assumptions that modeling choices bring with them (e.g., choice of specific distributions for parameters, constraints imposed by certain class of models like the Markovian assumption, heterogeneity of the data sources, and others). In our study, some of these drawbacks have been mitigated, for example by relaxing the markovian assumption (using time-dependent transition probabilities) and by eliciting preferences from a local population. For a more detailed discussion, along with recommendations for use, of the two approaches to cost-utility analysis we point the reader to the ISPOR Good Research Practices Report on the topic [28, 29].

The data presented in this study may suggest that TLM is superior to TORS with respect to C/U. However, the decision making on implementing a TORS or TLM program should be based also on additional objectives, such as the use of a robotic platform for endoscopic thyroid and neck surgery and/or other applications of the robot. While TLM is based on a technology platform less easy to expand, TORS uses technology for which new applications are easier to identify.

In summary, in this study we provide evidence for an advantage of TLM over TORS in terms of cost-effectiveness for the surgical minimally invasive treatment of operable OPSCCs. However, this advantage is sensitive to the rate of adjuvant treatment, the prescription of RT versus CRT, and the rate of patients requiring re-resections for inadequate margins.

## Data Availability

The datasets generated during and analyzed during the current study are not publicly available due to hospital policies but are available from the corresponding author on reasonable request. The datasets used and/or analyzed during the current study are available from the corresponding author on reasonable request.

## Footnotes

1 1 CHF currently corresponds to 0.96 Euro or 1.09 USD
